# Linking Histone Methylation States and *hsp* Transcriptional Regulation in Thermo-Tolerant and Thermo-Susceptible *A. mellifera* L. Subspecies in Response to Heat Stress

**DOI:** 10.3390/insects14030225

**Published:** 2023-02-24

**Authors:** Yehya Z. Alattal, Ahmad A. Alghamdi

**Affiliations:** Department of Plant Protection, College of Food and Agriculture Sciences, King Saud University, Riyadh 11451, Saudi Arabia

**Keywords:** *A. m. jemenetica*, *A. m. carnica*, ChIP, qPCR, histone methylation, epigenetic

## Abstract

**Simple Summary:**

Upregulation of heat shock proteins (*hsp*) is a key mechanism to diminish heat stress in *Apis mellifera*. In this study, we investigated how histone methylation states present an epigenetic layer of *hsp* regulation in response to heat stress in two honeybee subspecies: *A. m. jemenetica*, a thermo-tolerant honeybee subspecies native to the Arabian Peninsula, and *A. m. carnica*, a subspecies occurring naturally in temperate regions of Central Europe. The results revealed significant changes in most histone methylation states of *hsp* investigated. The results also revealed higher variation in histone methylation states associated with *hsp* in *A. m. carnica* compared to *A. m. jemenetica* after exposure to moderately high temperature. This confirms that the response threshold is lower for *A. m. carnica* compared to *A. m. jemenetica*. We concluded that changes in histone methylation states play a key role in regulation of *hsp* in both *A. mellifera* subspecies.

**Abstract:**

Genetic and epigenetic responses to environmental cues of worker honeybees mediate *hsp* synthesis, a key mechanism to tolerate high ambient temperatures in *Apis mellifera.* In this study, the chromatin immunoprecipitation assay followed by qPCR were used to determine alterations in histone methylation states (H3K27me2, H3K27me3, H3K4me2, and H3K4me3) associated with *hsp/hsc*/*trx* in *A. m. jemenetica* (thermo-tolerant subspecies) and *A. m. carnica* (thermo-susceptible subspecies) after heat treatment. The results revealed significant changes in enrichment folds of histone methylation states associated with *hsp/hsc*/*trx*. Indeed, the enrichment of H3K27me2 decreased strongly in response to heat stress. Changes in histone methylation states were significantly higher in *A. m. carnica* samples compared to *A. m. jemenitica* samples. Our study provides a new perception on linking histone post-translational methylation as an epigenetic mechanism of gene regulation with *hsp/hsc/trx* in *A. mellifera* subspecies exposed to heat stress.

## 1. Introduction

Heat shock protein (HSP) synthesis aims at keeping protein integrity and function, a well-documented molecular mechanism of thermoregulation in *Apis mellifera* [1,2,3]. On the basis of their molecular mass and function, HSPs are classified into six families: HSP20 (small HSPS), HSP40 (J-proteins), HSP60, HSP70, HSP90, and HSP100 [4]. Heat stress stimulates HSP synthesis and might cause aberrant DNA or histone post-translational modifications in many genes, providing a layer of epigenetic control over gene expression [5], a mechanism regulating cell responses to surrounding stimuli or stressors [2]. One of the key post-translational chromatin modifications is histone methylation [6]. Histone methylation is the modification of certain amino acids in a histone protein. It takes place by the addition of one (me), two (me2), or three (me3) methyl groups at lysine (K) residues of histones H3 and H4, providing different variants of histone marks including H3K4, H3K9, H3K27, H3K36, H3K79, and H4K20, which can be mono-, di-, or tri-methylated [6]. Histone methylation is catalyzed by histone methyltransferases (HMTs) and demethylases (HDMs); however, gene activation or silencing is resolved by deployed factors, including (i) modified amino acid position, (ii) methylation degree, and (iii) presence of specific enzymes; for example, H3K4me1 and H3K4me3 represent transcriptional active states, while H3K9me3 and H3K27me3 are repressive chromatin marks [7]. Histone methyltransferases (HMTs) and demethylases (HDMs) are antagonistic, and their abundance reflects the type of histone remodeling. A recent study reported extensive histone tail modifications in *Apis mellifera* by the employment of protein complexes and adding/removing of radicals to histone tails [8,9]. Furthermore, histone post-translational modifications (HPTMs) have been associated with female caste differentiation and behavior in *Apis mellifera* [8,10].

In addition to HSP upregulation, exposure of *Apis melifera* colonies to heat stress may trigger other different types of responses. As a eusocial structure, *Apis mellifera* colony exhibits unique behavioral responses to withstand such environmental variations [11]. This is especially valid for temperature cues that usually fluctuate in minutes to seasons. To avoid or withstand these fluctuations in ambient temperatures, the colony may start fanning [8,12,13,14,15], clustering [14,15], stinging, or even migratory swarming [12]. These behavioral responses are a sequence of steps that are regulated by genes and can include genetic/epigenetic modification of the individual honeybee [14,16]. Although an individual bee may not exhibit long-lasting thermoregulatory capabilities, the honeybee colonies of some *A. mellifera* subspecies can significantly diminish the impact of extreme temperatures under hot climates for a long duration [17,18]. Nowadays, *A. mellifera* comprises more than 30 geographical subspecies, occurring in very variable climatic zones [12,19]. Therefore, the honeybee colony of *A. mellifera* has been viewed as a super-organism and a model for functional homoeothermic insects [12,16,19,20]. Still, more investigations are required to better understand heat-resistance mechanisms of honeybees [17,21].

*A. m. jemenetica* is an indigenous subspecies of the Arabian Peninsula [12,22,23]. Existing literature reports that *A. m. jemenetica* was used in apiculture within this region from about 3500 years. It shows distinctive morphological and behavioral variations compared to other *A. mellifera* subspecies. It is the smallest and by far the most heat-tolerant honeybee subspecies compared with introduced subspecies such as *A. m. carnica*, *A. m. ligustica*, or *A. m lamarckii* [12,18,19]. In the central Arabian Peninsula, only *A. m. jemenetica* can persist [12,18,23,24].

In this article, histone methylation states (H3K4me2, H3K4me3, H3K27me2, H3K27me3) as epigenetic markers were associated with the relative profusion of ChIPed-DNA (*hsp*, *hsc70cb,* and *Apis mellifera* histone-lysine N-methyltransferase trithorax (trx)) and were compared with the indigenous honeybee subspecies of the Arabian Peninsula, *A. m. jemenetica* (thermo-tolerant) and *A. m. carnica* (thermo-susceptible subspecies that evolved in a temperate region) after exposure to heat stress. Changes in enrichment folds of different histone methylation marks suggest an epigenetic layer of gene regulation.

## 2. Materials and Methods 

### 2.1. Honeybee Sample Preparation

Four colonies of *A. m. jemenetica* were obtained from the King Saud University apiary (Riyadh, Saudi Arabia) to represent the indigenous heat-tolerant subspecies; four other colonies of *A. m. carnica* were assigned at Penn State University apiary (State College, PA, USA) as thermo-susceptible subspecies and were used later to collect samples. After standardization, colonies consisted of one brood box containing 10 frames of bee including 4–6 brood frames. Colonies were treated according to APIMONDIA guidelines of performance in both locations. In total, 16 samples of nurse-age bees were collected: 8 from colonies of *A. m. jemenetica* and 8 from colonies of *A. m. carnica*. Each sample consisted of 10 bees (in total, 160 bees were sampled). Four samples from each subspecies were assigned for control and four for treatment. Control and treatment samples were incubated for 1 h at 34 (control) and 42 °C (treated). Sugar water (1:1 *w*/*v*) was provided during incubation. Immediately after incubation, all individuals of each sample were nitrogen frozen. Thoraces of each sample were separated on a 100 mm plate; tissues were collected after unwanted parts such as necrotic materials were removed. Tissue samples were then weighed and macerated until no visible chunks were seen.

### 2.2. Cross-Linking, Quenching, and Chromatin Isolation

Tissues collected from each sample were cross-linked into a 15 mL conical tube using 1 mL cross-linking solution (270 µL of 37% formaldehyde) with 10 mL of DMEM culture medium (final concentration of formaldehyde was 1% per every 40 mg of tissue). Cross-linked tissues were then incubated at room temperature for 15–20 min on a rocking platform. Cross-linking reactions were quenched by adding 1 mL of 1.25 M glycine solution to every 9 mL of cross-linking solution. Then, they were mixed and centrifuged at 800 rpm for 5 min, the supernatant was discarded, and cells were then washed three times with 10 mL of ice-cold phosphate buffer solution (PBS), once with centrifugation at 800 rpm for 5 min, and the supernatant was discarded. In total, 16 samples were prepared (eight *A. m. carnica* samples: four treated (CC) and four control (CT); eight *A. m. jemenetica* samples: four treated (YT) and four control (YC)). Each pooled sample was then homogenized and subjected to chromatin isolation using a ChromaFlash^TM^ Chromatin Extraction Kit (P-2001) according to the kit instructions (total volume of chromatin solution: 200 µL/sample). Extracted chromatin was sheared using the Episonic2000 Sonication System. The sheared chromatin concentration was measured by fluorescence quantification using a Qubit 4 Fluorometer^®^ (Thermofisher, Waltham, MA, USA)—200 µL of chromatin solution was used per sample.

### 2.3. Chromatin Immunoprecipitation (ChIP)

Pooled samples were then subjected to chromatin immunoprecipitation (ChIP). For immunoprecipitation, 6 µg of K4me2, K4me3, K27me2, or K27me3 antibodies (#A-4032, #A-4033, #A-4038, #A-4039, respectively; Epigentek, Farmingdale, NY, USA) were combined with 12 µL of ChIP assay beads, 3 µg of chromatin solution, and 500 µL ChIP assay buffer in a 1.5 mL tube [25]. As negative control of 6 µg of non-immune IgG was used. The samples were incubated at room temperature for 180 min with continuous rotation. After incubation, the beads were washed, and the ChIPed DNA was purified and eluted using 60 µL of water. A total of 1 µL oF purified DNA was used for quantification of ChIPed DNA using a Qubit 4 Fluorometer^®^ (Thermofisher, Waltham, MA, USA). DNA was purified using 10 µL of sheared chromatin in the case of *A. m. carnica* and 20 µL in the case of *A. m. jemenetica*.

### 2.4. Quantitative Polymerase Chain Reaction (qPCR)

Quantitative polymerase chain reaction (qPCR) was then performed using 1 µL of the purified DNA and gene-specific primers designed for the target gene region (see below, Table 1). The 7500 Real-Time PCR System (Applied Biosystems: ABI), 96-well PCR plates (ABI), and optical quality sealing tapes (ABI) were used for qPCR. DNA from non-immune IgG was used as negative control for determining the enrichment fold. Un-ChIPed DNA (5% for *A. m. carnica* samples and 10% for *A. m. jemenitica* samples) was used as input for determining enrichment efficiency (Input%). Then, data analysis was performed using qPCR Ct values to calculate enrichment fold and input% for each target region (enrichment fold = 2^ct(IgG-AB)^, input % = 2^ct (Input-AB)−(dilution factor)^ × 100%). Dilution factors were 20-fold (5%) for CC and CT and 10-fold (10%) for YC and YT. The dilution factor was used to normalize input DNA to 100%. Analyses of enrichment fold values between treated and control samples were performed using GraphPad Prism 8.0.1. (www.graphpad.com, accessed on 2 February 2023). Significant variation was determined using the multiple *t*-test with the two-stage linear step-up procedure of Benjamini, Krieger, and Yekutieli (*p* < 0.05 and Q = 0.01); each methylation mark was analyzed individually without assuming a consistent SD, and the total number of tests per gene was four.

### 2.5. Heat Shock Protein Primer Design

The public databases Genbank (NCBI, Honeybee Genome Consortium) were mined for known small- and large-molecular-weight HSP and trxG. The genes included HSP10, HSP28, HPS60, HSP70, HSP83, and HSP90 and histone-lysine N-methyltransferase (NCBI, Honeybee Genome Consortium, 2008). The gene sequences were downloaded into Geneious^®^ Prime 2021.1.1 (https://www.geneious.com, accessed on 1 May 2021) for analysis and primer design (Table 1).

## 3. Results

**Figure 1:**
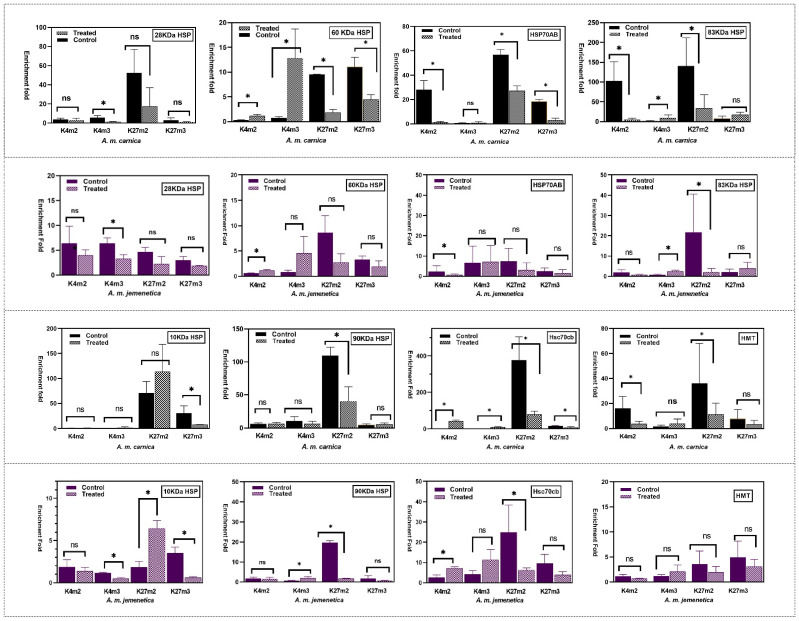
Comparison of the ChIP-qPCR enrichment folds of four histone marks at many heat shock protein genes, histone-lysine N-methyltransferase trithorax (*trx*), and heat shock cognate 70 cb (Hsc70cb) in thermo-tolerant *A. m. jemenetica* and thermo-susceptible *A. m. carnica* after exposure to heat stress (42 °C for one hour). Active marks antibodies (H3K4me2 and H3K4me3) and repressive marks (H3K27me2 and H3K27me3) were used to pull down sonicated chromatin. DNA from non-immune IgG was used as a negative control to calculate the enrichment fold. Enrichment fold = 2^ct(IgG-AB)^. (*) refers to significant differences between treatment and control, (ns) refers to non-significant differences (Table S1).

The results stated that changes in the histone methylation/demethylation states (H3K27 and H3K4) after heat treatment were associated with *hsp/hsc*/*trx* transcript abundance primed for expression in both honeybee subspecies. Decreased deposition of the repressive mark H3K27me2 was distinctly a key change in response to heat treatment. The treatment’s enrichment changes were much higher in *A. m. carnica* (the thermos-susceptible subspecies) compared to *A. m. jemenetica* (the thermo-tolerant subspecies) at 42 °C (Figure 1). However, the amount of deposition of H3K27me2 in *A. m. carnica* control samples was in many cases 10× higher than that of *A. m. jemenetica* (Figure 1). In the case of *A. m. carnica*, variations in enrichment folds between the control and treated samples were significant in comparison of most methylation states (H3K4me2: *hsp60*, *hsp70ab*, *hsp83*, *hsc70cb,* and *trx*; H3K4me3: *28hsp*, *hsp60*, *hsp83,* and *hsc70cb*; H3K27me2: *hsp60*, *hsp70ab*, *hsp83*, *hsp90*, *hsc70cb*, and *trx*; H3K27me3: *hsp60*, *hsp70ab*, *hsp10*, and *hsc70cb)* (Figure 1).

In the case of *A. m. jemenetica*, histone methylation enrichment folds in control samples were generally lower compared to *A. m. carnica* control samples. Nonetheless, differences between control and treated *A. m. jemenetica* samples were significant in several methylation states (H3K4me2: *hsp60*, *hsp70ab*, and *hsc70cb*; H3K4me3: *28hsp*, *hsp83*, *hsp90,* and *hsp10*; H3K27me2: *hsp60*, *hsp83*, *hsp10*, *hsp90,* and *hsc70cb*; H3K27me3: *hsp10* only) (Figure 1). Apparently, the highest change in histone methylation enrichment folds occurred for H3K27me2 at *hsc70cb* in both honeybee subspecies. In the case of *Apis mellifera* HMT (histone-lysine N-methyltrans-ferase trithorax (*trx*)), variations in enrichment folds of all methylation states (H3K4me2, H3K4me3, H3K27me2, and H3K27me3) were insignificant for all methylations states between the treated and control *A. m. jemenetica* samples.

## 4. Discussion

This is the first study reporting on variation in methylation/demethylation states of histone H3 lysine K4 and K27 as an epigenetic modification associated with heat treatment in *Apis mellifera*, a mechanism that retained suppression/activation of *hsp* gene transcription. Obviously, low deposition of H3K27me2 is the main consistent change among the four histone methylation states (H3K27me2, H3K27me3, H3K4me2, and H3K4me3) that is associated with profusion of the targeted genes (*hsp*, *hsc70cb,* and *trx*) in response to heat stress in both honeybee subspecies (Table S1).

*Hsc70cb* had the highest reduction in enrichment folds of H3K27me2 in both honeybee subspecies, demonstrating an important role for *hsc70cb* in response to heat stress in *A. mellifera.* Previous studies have indicated that hsc79cb is an effective suppressor of protein misfolding and optimally acts as an NEF for Hsp70 [26,27,28,29]. Moreover, significant variations in enrichment folds of different histone methylation marks occurred at large- (*hsp60*, *hsp70ab*, and *hsp90*) as well as small (*hsp28* and *hsp10*)-molecular-weight HSP, indicating that response to heat stress in *A. mellifera* can involve several heat shock proteins. A previous study [30] reported Hsp70 from *A. m. jemenetica* samples exposed to heat stress. Yet, the study [30] may not have been able to detect other HSPs in its treated samples due to the employed methodology and experimental conditions. It can be expected that long periods of temperature extremes are associated with the expression of many interlaced HSPs.

In this study, the relatively higher variation in deposition of the four histone methylation states between the control and treated *A. m. carnica* samples indicate higher sensitivity of this honeybee subspecies to the used temperature. On the contrary, the relatively small changes in the deposition of the four histone marks (H3K27me2, H3K27me3, H3K4me2, and H3K4me3), *hsp*, *hsc70cb,* and *trx* in the case of *A. m. jemenitica*, might be related to several factors, including a higher response threshold as a thermo-tolerant subspecies (in this study, we exposed the samples to 42 °C for one hour, which may not be enough to induce higher response in *A. m. jemenetica*), and/or to the smaller body size of *A. m. jemenetica* compared to *A. m. carnica*, as was documented in the case of stingless bees, where bee body size was positively correlated with *hsp* expression rates [31,32]. Nevertheless, in isothermic insects such as *D. melanogaster*, the variation in histone marks in response to heat treatment was relatively higher compared to *A. mellifera* as heterothemic insect [33]. For cosmopolitan species (such as *A. mellifera*) naturally occurring in different thermal gradients, populations/ecological subspecies may exhibit different thermal responses to extreme temperatures [12,34,35]. This increases local adaptation of populations inhabiting warmer climates and leads to better fitness of these populations at higher temperatures relative to populations from temperate areas and vice versa [36]. A long-term study [18] reported lower performance and survival rates of *A. m. carnica* colonies under Saudi Arabian conditions. However, thermo-tolerance of *A. m. jemenetica* may be diminished with the intensification of climate change [37]. Therefore, determination of heat-resistance thresholds of different honeybee subspecies would be very useful. Furthermore, quantitative investigation of HSP expression dynamics in real time associated with different epigenetic control mechanisms will increase our understanding of the molecular aspects of heat tolerance in both honeybee subspecies.

In conclusion, this study presents evidence on the association between histone post-translational methylation and *hsp* transcript abundance in *Apis mellifera* exposed to heat treatment. It is also obvious that variation in enrichment folds of H3K27me2 of different *hsp* forms the key response to heat treatment.

## Figures and Tables

**Table 1:** Primer sequences with gene names, gene subcellular location, and NCBI gene ID for *hsp*, *hsc/trx* genes used in the present study.

Locus/Gene Identifier	Gene/DNA Region	Primers
LOC724487	28 KDa heat- and acid-stable phosphoprotein-like	F- GAGGAACCCAAAGCACATGGT R- TCTACACCCTTTGTTTTTCCCTGT
LOC552531	10 KDa heat shock protein, mitochondrial-like	F- AGCAATTGGACCTGGACAAAGA R- GCCAGTATATCTGACTCACGGAAT
LOC409384	60 KDa heat shock protein, mitochondrial-like	F- CCACGCCTGCATTTTGAGCA R- GCAACCAGAGCAGCCGTTGA
LOC410620	Heat shock protein 70Ab	F- TGGCATTCCACCTGCACCTA R- TGGTGATCTTGTTCTCCTTTCCAGT
LOC408706	Heat shock cognate 70Cb ortholog	F- CGCGCGTCTACACGTTCTTT R- CGTGATTTTGATGCCGCAGT
LOC411700	Heat shock protein 83-like	F- TCCACATCTTCTGCTTTTGTTTCC R- TCAACGCGCGTCTTCATTCA
LOC408928	Heat shock protein 90	F- TGGATCCGTGAGAGATTCATAGCG R- CGCTTTCCAAGCTGAAATTGCACA
XM_006559101.1	*Apis mellifera* histone-lysine N-methyltrans-ferase trithorax (trx), transcript variant X4,	F-TGCAGCTAGATTCATTAATCATTCATG R-CATGGAATCTTGATATCCTCGAAAG

## Data Availability

Data are available in tables and figures.

## Supplementary Materials

The following are available online at https://www.mdpi.com/article/10.3390/insects14030225/s1, Table S1: Difference ± SE of enrichment folds of different histone marks at different target genes with the associated P and Q values determined using the Two-stage linear step-up procedure of Benjamini, Krieger and Yekutieli, without assuming a consistent SD.
