# Supplementary material for: Linking Histone Methylation States and hsp Transcriptional Regulation in Thermo-Tolerant and Thermo-Susceptible A. mellifera L. Subspecies in Response to Heat Stress
Source: Insects. 2023 Feb 24;14(3):225. doi: 10.3390/insects14030225 (PMC10057246; doi:10.3390/insects14030225)
Supplement: Supplementary file 1 [file insects-14-00225-s001.zip › insects-2232370-supplementary.pdf]

Table S1. Difference±SE of enrichment folds of different histone marks at different target genes with the associated P and Q values determined using the Two-stage linear step-up procedure of Benjamini, Krieger and Yekutieli, without assuming a consistent SD.

Histone mark	Gene name	JEMENETICA			CARNICA		
		Difference±SE	P value	q value	Difference±SE	P value	q value
K4m2	60KDa HSP	-0.5350±0.09046	0.004093	0.033072	-0.8700±0.1535	0.004775	0.009919
K4m3	60KDa HSP	-3.749±1.914	0.121716	0.282304	-12.10±3.388	0.023339	0.028007
K27m2	60KDa HSP	5.889±1.805	0.030982	0.125166	7.670±0.3499	0.000026	0.000123
K27m3	60KDa HSP	1.384±0.7525	0.139755	0.282304	6.645±1.260	0.006200	0.009919
K4m2	90KDa HSP	0.1869±0.5867	0.766039	0.459623	-0.2850±1.635	0.870095	0.878796
K4m3	90KDa HSP	-1.184±0.3867	0.037617	0.045140	4.475±4.031	0.329186	0.664956
K27m2	90KDa HSP	18.48±0.03485	<0.000001	<0.000001	69.80±14.58	0.008733	0.035281
K27m3	90KDa HSP	1.054±0.7839	0.250023	0.200018	-1.025±1.550	0.544458	0.733204
K4m2	28KDa HSP	2.371±2.097	0.321538	0.096461	0.5350±1.452	0.731120	0.658008
K4m3	28KDa HSP	3.119±0.7860	0.016567	0.019880	4.280±1.380	0.036199	0.130317
K27m2	28KDa HSP	2.399±0.9961	0.073688	0.029475	33.21±17.63	0.132776	0.221984
K27m3	28KDa HSP	1.137±0.4255	0.055664	0.029475	2.125±1.329	0.184987	0.221984
K4m2	83KDa HSP	1.273±0.7620	0.170231	0.136184	96.04±28.55	0.028268	0.045229
K4m3	83KDa HSP	-1.843±0.3964	0.009668	0.018044	-12.98±2.624	0.007791	0.014684
K27m2	83KDa HSP	25.80±6.315	0.015037	0.018044	130.4±30.04	0.012237	0.014684
K27m3	83KDa HSP	-2.009±1.913	0.352912	0.211747	-7.298±4.095	0.149254	0.105782
K4m2	70APKDa HSP	2.100±1.563	0.250139	0.811305	26.74±4.326	0.003480	0.008353
K4m3	70APKDa HSP	-0.2500±6.186	0.969699	>0.999999	-0.3900±0.5103	0.487318	0.292391
K27m2	70APKDa HSP	4.450±4.093	0.338044	0.811305	27.57±2.957	0.046228	0.055473
K27m3	70APKDa HSP	0.8000±1.347	0.584588	0.935341	12.48±5.080	0.013466	0.008080
K4m2	NMT	0.4436±0.2170	0.110439	0.502852	14.73± 3.240	0.010434	0.050083
K4m3	NMT	-0.9212±0.7716	0.298518	0.502852	-3.583±1.330	0.054370	0.086992
K27m2	NMT	1.592±1.651	0.389426	0.502852	32.70±11.05	0.041592	0.086992
K27m3	NMT	1.828±2.031	0.419043	0.502852	5.700±2.598	0.093249	0.111899
K4m2	70cp like	-4.680±0.8549	0.005419	0.021891	-45.56±0.9382	0.000001	0.000003
K4m3	70cp like	-7.030±3.119	0.087246	0.114462	-7.495±1.389	0.005701	0.008636
K27m2	70cp like	14.03±4.406	0.033417	0.067502	305.6±74.68	0.014943	0.015093
K27m3	70cp like	5.545±2.743	0.113329	0.114462	8.365±2.287	0.021639	0.016391
K4m2	10KDa-HSP	-0.06667±0.4028	0.876563	0.885328	0.4820±0.5328	0.416789	0.315718
K4m3	10KDa-HSP	-1.000±1.180	0.444443	0.598517	0.6732±0.08052	0.001119	0.003390
K27m2	10KDa-HSP	-42.70±34.28	0.280941	0.567500	-4.609±1.697	0.053207	0.080609
K27m3	10KDa-HSP	21.93±8.661	0.064496	0.260564	4.186±2.825	0.212619	0.214746
